# 3D Non-Woven Polyvinylidene Fluoride Scaffolds: Fibre Cross Section and Texturizing Patterns Have Impact on Growth of Mesenchymal Stromal Cells

**DOI:** 10.1371/journal.pone.0094353

**Published:** 2014-04-11

**Authors:** Anne Schellenberg, Robin Ross, Giulio Abagnale, Sylvia Joussen, Philipp Schuster, Annahit Arshi, Norbert Pallua, Stefan Jockenhoevel, Thomas Gries, Wolfgang Wagner

**Affiliations:** 1 Stem Cell Biology and Cellular Engineering, Helmholtz-Institute for Biomedical Engineering, RWTH Aachen University Medical School, Aachen, Germany; 2 Institute for Textile Technology RWTH Aachen University, Aachen, Germany; 3 Department of Plastic and Reconstructive Surgery, Hand Surgery, Burn Center, RWTH Aachen University, Aachen, Germany; 4 Department of Applied Medical Engineering, Helmholtz-Institute for Biomedical Engineering, RWTH Aachen University, Aachen, Germany; Institute for Frontier Medical Sciences, Kyoto University, Japan

## Abstract

Several applications in tissue engineering require transplantation of cells embedded in appropriate biomaterial scaffolds. Such structures may consist of 3D non-woven fibrous materials whereas little is known about the impact of mesh size, pore architecture and fibre morphology on cellular behavior. In this study, we have developed polyvinylidene fluoride (PVDF) non-woven scaffolds with round, trilobal, or snowflake fibre cross section and different fibre crimp patterns (10, 16, or 28 needles per inch). Human mesenchymal stromal cells (MSCs) from adipose tissue were seeded in parallel on these scaffolds and their growth was compared. Initial cell adhesion during the seeding procedure was higher on non-wovens with round fibres than on those with snowflake or trilobal cross sections. All PVDF non-woven fabrics facilitated cell growth over a time course of 15 days. Interestingly, proliferation was significantly higher on non-wovens with round or trilobal fibres as compared to those with snowflake profile. Furthermore, proliferation increased in a wider, less dense network. Scanning electron microscopy (SEM) revealed that the MSCs aligned along the fibres and formed cellular layers spanning over the pores. 3D PVDF non-woven scaffolds support growth of MSCs, however fibre morphology and mesh size are relevant: proliferation is enhanced by round fibre cross sections and in rather wide-meshed scaffolds.

## Introduction

Mesenchymal stromal cells (MSCs) raise high expectations in regenerative medicine. They can easily be expanded *in vitro*, comprise a subset with multilineage differentiation potential often referred to as “mesenchymal stem cells”, and they have immunomodulatory properties [1]–[4]. Usually, MSCs are culture expanded on tissue culture plastic (TCP) – particularly on 2D polystyrene surfaces. For therapeutic applications the cells are then harvested and injected in suspension. However, tissue engineering of complex lesions or interventions aimed at repairing hierarchically organized tissues requires the implantation of cells in a suitable scaffold which facilitates 3D cell integration [5]–[7].

A multitude of biomaterials has been used in tissue engineering. Hydrogels and fibrous scaffolds based on synthetic or natural polymers were shown to be suitable for MSC expansion [8]–[10]. Fibre based structures represent a promising approach for tissue engineering due to their close resemblance to native extracellular matrix (ECM)[11]. Such fibre based structures have large surface areas and porosity that can be adjusted to the specific cellular requirements [12]. Hence, these biomaterials are intensively studied for numerous applications in tissue engineering, including ligament repair [13], [14], bone and cartilage regeneration [15], [16] and soft tissue replacement [17], [18]. Depending on the application it is advantageous to either use a biodegradable material which is resorbed in the course of tissue regeneration and restructuring, or to rather use a non-biodegradable material if long-term stability and functionality is required. Non-degradable meshes of polymers can be used to coat a broad range of implants. Vascular stents, for example, have to remain attached and integrated into the surrounding tissue to keep the lumen permanently opened. Various biostable polymers such as polyethylene terepthalate (PET), have been used to coat stents [19].

Polyvinylidene Fluoride (PVDF) has been used as suture material [20], [21], for the construction of hernia meshes [22], [23] and for mechanical supporting meshes in vascular tissue engineering [24]. It provides good biocompatibility, is biologically inert, non-toxic, non-degradable and resistant to bacterial infections [25]. In contrast to other polymers such as polypropylene (PP) or PET, PVDF shows less inflammatory reactions, less fibrotic tissue formation and it is more resistant to hydrolysis and degradation [20]–[23]. Furthermore, monofilament sutures out of PVDF reveal very good long-term stability under tension and can be sterilized by beta or gamma radiation [26].

Previous studies demonstrated that surface patterning and 3D composition of tissue engineering scaffolds have major impact on cellular behavior [27]. Under *in vivo* conditions, the extracellular microenvironment (e.g. basement membrane and ECM) is not flat, but rather arranged in semi-aligned sheets with grooves, ridges and pores [28], [29]. It has been shown that mechanical cues such as micro-patterns and substrate elasticity influence cell growth and differentiation [30], [31]. In fact, various cell types such as MSCs, osteoblasts, fibroblasts and endothelial cells align, elongate and migrate along structured surfaces [32]. Non-woven structures which are mechanically bonded together by entangling fibres resemble some of the characteristics of ECM and may therefore be promising scaffolds for tissue engineering [33], [34]. So far little is known about the effect of fibre cross section geometry or pore architecture on MSCs growth and integration.

In the present study, highly porous PVDF non-wovens were produced with varying fibre cross section and crimp and subsequently used as scaffold for MSCs. We demonstrate that MSCs adhere and proliferate better on non-wovens with round fibre cross section, although the surface area on trilobal and snowflake cross section is significantly larger. Electron microscopy revealed that MSCs form small layers spanning over the non-woven pores. Overall, PVDF non-wovens support MSCs growth and differentiation and therefore represent suitable alternatives for tissue engineering.

## Material and Methods

### Ethics statement

MSCs from adipose tissue were isolated after patient's written consent using guidelines approved by the Ethic Committee of the University of Aachen (Permit number: EK163/07).

### Manufacturing of PVDF non-woven scaffolds

**Figure 1:**
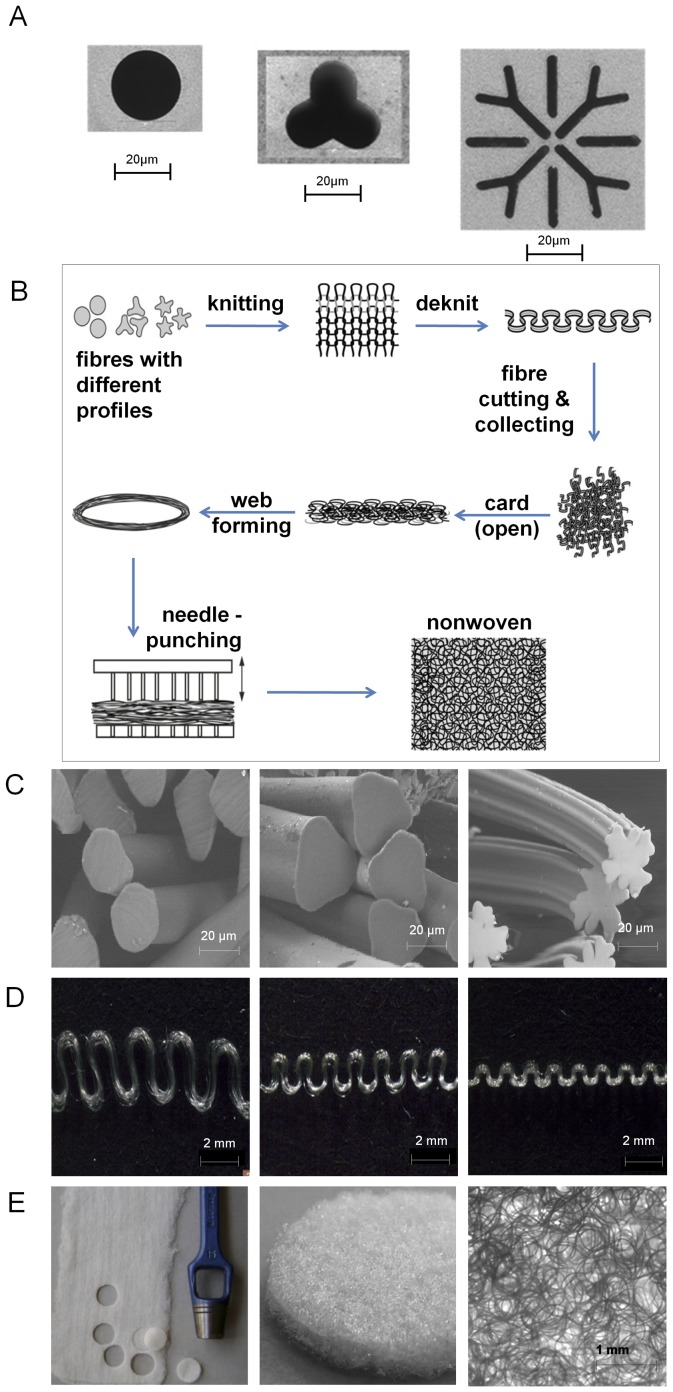
Manufacturing steps of three-dimensional PVDF non-wovens. Round shaped (f24 0.4 L/D 2, left), trilobal (Y24 250×552 L/D 2, middle), and snowflake (f24 L/D 2, right) spinnerets (A). Schematic overview of the fabrication process of non-wovens made of PVDF fibres (B). SEM pictures of round (left), trilobal (middle) and snowflake (right) non-wovens cross-sections (C). Fibre texturizing of fibres knitted with 10 (left), 16 (middle), or 28 (right) needles/inch (D). Round scaffolds are punched out of the non-woven fabrics (E).

Polymeric PVDF granules (PVDF Solef 1006 by Solvay Solexis S. A., Tavaux, France) were processed into multifilament fibres with 24 single fibres using the coextrusion spinning plant (Fourné Polymertechnik GmbH, Germany) in a single extrusion mode. Besides round shaped filaments also trilobal and snowflake-shaped fibres were produced. The respective spinneret geometries are shown in Figure 1A–C. A Fully-Drawn-Yarn (FDY) Take Up with one godet-duo and two heatable single godets were used. The temperature profile of the spin line was set as follows: 1^st^ extruder zone 235°C, 2^nd^ extruder zone 240°C, 3^rd^ extruder zone 240°C, melt pipe 245°C, melt pump 250°C, spinning head 245°C. As spin finish Dryfi PP I (Schill+Seilacher GmbH, Germany) was used. For subsequent processing steps Silastol R641 (Schill+Seilacher GmbH, Germany) was applied to the PVDF fibres.

The production of non-woven fabrics consists of three main processing steps: texturing, web formation, and bonding (Figure 1B). Starting point for the non-woven process is the multifilament yarn mentioned above. To obtain a stable non-woven structure the yarn must be textured. A texturing-method which produces a homogeneous and reproducible wavelike texture is called “knit-deknit”. Therefore, we used a circular knitting machine (TK 83 of Harry Lucas Textilmaschinenfabrik, Neumünster, Germany) with various knitting parameters to adjust the amplitude and the wavelength of the texture. A permanent shape was facilitated by thermal fixation the yarn at 120°C for 5 minutes. After cutting the multifilament into staple fibres, the cut fibre bundles were separated into single fibres. This “opening process” was carried out by the lab-scaled card MDTA3 (Zellweger, Uster, Swiss) and repeated three times to obtain a homogenous web. Subsequently, this web was mechanically bonded by using a lab-scaled needle-punching machine designed at the Institute for Textile Technology Aachen (ITA). For the needle-punching, 15×18×40×3 FBD6 needles (Groz-Beckert, Albstadt, Germany) were selected. Finally, round scaffolds with a diameter of 15 mm were manually punched out of the non-woven fabrics (Figure 1E).

For production of non-structured, even PVDF substrates 1.5 g PVDF granules were melted at 200°C for 5 min and subsequently pressed for 1 min at 150°C. The PVDF were subjected to hydrogen peroxide gas plasma treatment in a plasma sterilizer (Sterrad 100 s, Johnson & Johnson, Brunswick, NJ, USA). The duration of the plasma treatment was 7 min (505 mTorr).

### Isolation and characterisation of MSCs

MSCs were isolated from lipoaspirates from human healthy donors. In brief, lipoaspirates were washed in 9 g/L NaCl, centrifuged at 300 *g* for 10 minutes and the middle layer was subsequently digested with 2 g/L collagenase type I (Biochrom, Berlin, Germany) and with 15 g/L BSA (PAA, Pasching, Austria) for 45 min at 37°C under constant shaking. The digested tissue was passed through a 100 µm cell strainer and seeded in culture flasks (Nunc Thermo Fisher Scientific, Langenselbold, Germany). Culture medium consisted of Dulbecco's Modified Eagles Medium-Low Glucose (DMEM-Low Glucose; PAA, Pasching, Austria) with 2 mM L-glutamine (Gibco/Invitrogen, Eugene, OR, USA), 100 U/mL penicillin/streptomycin (Gibco/Invitrogen, Eugene, OR, USA) and 10% human platelet lysate (HPL) which was pooled from five platelet units of healthy donors as described before [35]–[37]. Cultures were maintained at 37°C in a humidified atmosphere containing 5% CO_2_ with medium changes twice per week. MSCs were always harvested by trypsinization upon 80% confluent growth, counted with a Neubauer counting chamber (Brand, Wertheim, Germany) and re-seeded in a density of 10,000 cells/cm^2^ in 75 cm^2^ culture flasks. Immunophenotypic analysis of various surface markers (CD14, CD29, CD31, CD34, CD45, CD73, CD90, and CD105) and *in vitro* differentiation potential towards adipogenic and osteogenic lineages was validated for cell preparations as described in our previous work [38].

### Seeding of MSCs on non-wovens

Non-wovens were placed in 6-well plates and disinfected by ethanol treatment. MSCs at passage 3 were then harvested by trypsinisation and about 19,000 cells were seeded with a pipette on PVDF non-wovens with a surface of 1.9 cm^2^. Culture conditions were then used as described above with medium changes twice per week.

### Proliferation analysis

Proliferation of MSCs on flat PVDF surface was quantified by cell counting: MSCs were seeded at a density of 10,000/cm^2^ and cells were counted at day 5 with a Neubauer counting chamber with Trypan blue exclusion (3 technical replicas per condition). Cell population doublings (PDs) within 5 days were then estimated by the ratio of seeded *versus* harvested cells. Since not all cells can be harvested from the 3D non-wovens we have estimated proliferation within these scaffolds using the Alamar Blue assay according to manufacturer's instructions (Invitrogen, Eugene, OR, USA) [39]. Cells were analysed at day 1, 5, 10 and 15 after seeding on the non-wovens. To exclude cells from the underlying TCP, the nonwovens were always transferred into a fresh well and then incubated with 1 ml culture medium containing 1×10^−4^ M resazurin (Invitrogen, Eugene, OR, USA) for 5 h at 37°C. The medium was removed from the scaffolds and fluorescence was measured with a Tecan Infinite 2000 plate reader at excitation and emission wavelengths of 560 nm and 590 nm, respectively.

### Adipogenic and osteogenic differentiation

Adipogenic differentiation was induced by culture medium consisting of DMEM (PAA) containing 10% HPL, 0.5 mM isobutylmethylxanthine (IBMX; Sigma, St. Louis, MO, USA), 1 µM dexamethasone (Sigma) and 10 µM insuline (Sigma) as described before [40], [41]. Osteogenic differentiation medium consists of DMEM-low glucose (PAA) with 2 mM *L*-glutamine (Sigma), 100 U/mL pen/strep (Lonza), 100 nM dexamethasone (Sigma), 200 µM *L*-ascorbic acid-2-PO_4_ (Sigma), 10 mM β-glycerophosphate (Sigma). For staining of lipid droplets we initially tried the conventional staining method with Oil red but this dye has high affinity for the PVDF-substrates. Therefore, we stained fat droplets with the green fluorescent dye BODIPY (4,4-difluoro-1,2,5,7,8-pentamethyl-4-bora-3a,4a-diaza-s-indacene) counter-stained with DAPI (4′,6-Diamidin-2-phenylindol; both Molecular Probes, Eugene, Oregon, USA). Fluorescence microscopy pictures were taken from representative areas. Images acquisition was performed using a Leica DM IL LED microscope (Leica, Wetzlar, Germany) with a 10x dry objective (numerical aperture: 0.3; Leica) and a camera (Leica DFC420C) equipped with Leica application suite 3.3.1 software. For analysis of calcium deposits upon osteogenic differentiation we tested Alizarin Red as described before [42].

### Quantitative RT-PCR analysis

In addition, we validated adipogenic differentiation by increased gene expression of adiponectin (*ADIPOQ*), fatty acid binding protein 4 (*FABP4*) and peroxisome proliferator-activated receptor γ (*PPARy*) after two weeks as described before [43]. Osteogenic differentiation was also addressed by the expression of alkaline phosphatase (*ALP*), runt-related transcription factor 2 (*RUNX2*), osteocalcin (*BGLAP*), and osterix (*OSX*). For each PVDF non-woven we have also analyzed MSCs without induction of differentiation. Expression levels were calculated for each sample in relation to *GAPDH* (ΔCT) [44]. To determine up-regulation of differentiation markers the expression levels were subsequently normalized to the corresponding undifferentiated controls (ΔΔCT).

### Scanning electron microscopy

MCSs seeded on PVDF non-wovens were fixed in 3% glutaraldehyde for at least 1 hour at room temperature, rinsed with PBS and dehydrated by serial incubations in 30%, 50%, 70%, 90%, and 100% ethanol for 10 minutes at room temperature. The compounds were then critical point dried in liquid CO_2_ and sputter-coated with a 30 nm gold layer using an ion sputter coater (LEICA EM SCD 500). Samples were analysed using an environmental scanning electron microscope at the electron microscope facility, RWTH Aachen University (ESEM XL 30 FEG, FEI, Philips, Eindhoven, Netherlands).

### Statistics

Results are expressed as mean ± standard deviation of at least three independent experiments. To estimate the probability of differences we have adopted the paired two-sided Student's T-test.

## Results

### PVDF is a suitable biomaterial for MSCs

Before analysing MSC growth in 3D non-woven PVDF scaffolds we performed preliminary experiments with 2D PVDF-surfaces in comparison to normal polystyrene tissue culture plastic (TCP). MSCs did proliferate on PVDF substrates although the proliferation rate – estimated by measuring the population doublings (PDs) within five days – was significantly higher on conventional TCP. We compared two different PVDF preparations, PVDF1006 and PVDF1008 – the latter characterized by lower viscosity and higher molecular weight (Solvay, Solexis 2006). No differences in MSC growth were observed between PVDF1006 and PVDF1008 (6.2 PDs, 4.7 PDs, and 4.9 PDs within 5 days on TCP, PVDF1006 and PVDF1008, respectively). Therefore we have used PVDF1006 for all subsequent experiments (Figure S1A). Processing of multifilament PVDF fibres requires a thin oil film coating step to prevent static charging. We considered that residual traces of oil might interfere with MSC growth on PVDF non-wovens, thus we analysed MSC growth on PVDF substrates treated with two distinct oil preparations (R641 and PP1) followed by three additional washing steps with PBS and either with or without additional washing steps with ultrasonic treatment. Overall, MSCs displayed similar proliferation rates on PVDF with different oil preparations and washing procedures (ranging from 4.5 PDs to 4.9 PDs within 5 days) indicating that in our experimental setting residual oil traces are either not present or do not have major impact on cell growth (Figure S1B). Alternatively, the PVDF substrates were subjected to plasma treatment to promote cell attachment and proliferation [45], [46] but this did not increase MSC proliferation and therefore this approach was not adopted for the following experiments (Figure S1C). Taken together, 2D-PVDF is a suitable biomaterial to support growth of MSCs, but the proliferation rates are significantly lower than on conventional TCP.

### Seeding efficiency of MSCs into PVDF non-wovens

Afterwards, we generated PVDF fibres with different cross-sectional shape (round, trilobal and snowflake). The draw-ratio (ratio between draw-off godet and feed-godet), fineness of the multifilament yarn, single fibre fineness, mechanical characteristics of tensile strength and elongation at break point of the as-spun fibres are shown in Table 1. Each of these fibres was further texturized by knit-de-knit procedures using 10, 16 or 28 needles/inch resulting in nine different well characterized PVDF non-wovens with different values of pore size and pore number (Table 2)(Figure 1D). The average fibre circumference varied significantly according to the different fibre profiles: 79.4 µm for round fibres, 96.6 µm for trilobal fibres and 150.8 µm for snow flake fibres. The average non-woven weight ranged from 61.3 mg to 74.7 mg with an average non-woven thickness of 2.8 mm (±0.2 mm).

**Table 1:** Fibre Characteristics.

	draw-ratio	fineness of the multifilament yarn [dtex]	single fibre fineness [dtex]	single fibre fineness [µm]*	tensile strength [cN/dtex]	elongation at break [%]
round	2	180.9	7.5	23	2.3	61.1
trilobal	2.5	257.6	10.7	41	2.1	90.9
snowflake	2	175.7	7.3	29	2.1	39.9

*Mean single fibre fineness [µm] was calculated for the different fibre profiles by deviation of the ideally round fibre profile.

**Table 2:** Characterisation of non-wovens properties.

	pore size [µm]	standard deviation [µm]	pore number	standard deviation
round 10	246	19	107	95
round 16	192	84	93	151
round 28	203	15	80	31
trilobal 10	213	14	208	35
trilobal 16	229	13	82	94
trilobal 28	224	12	140	170
snow flake 10	211	7	226	107
snow flake 16	246	22	125	155
snow flake 28	254	26	83	38

**Figure 2:**
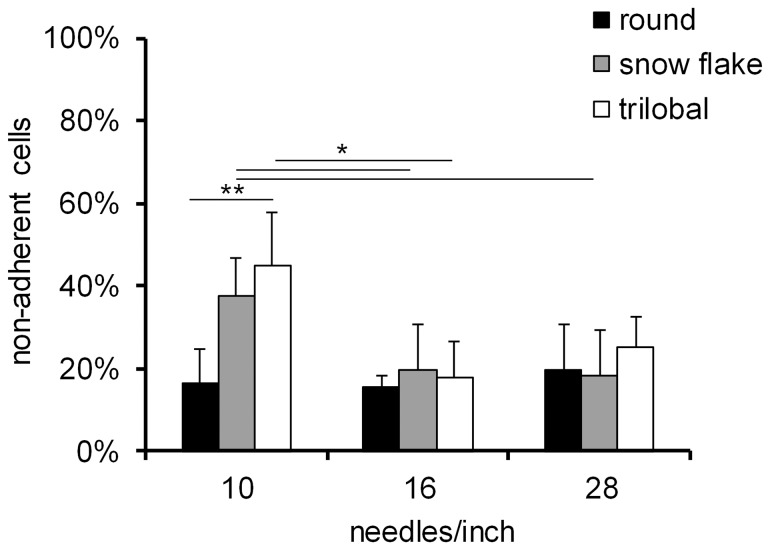
Cell loss upon initial seeding in PVDF non-wovens with different fibre texturizing. Percentage of non-adherent cells was determined by cell counting 15 min after seeding (n = 3). *p<0.05; **p<0.005.

These parameters might affect the seeding efficiency and the capability of MSCs to adhere to the scaffolds. To address this question, MSCs were seeded on the non-wovens by pipetting: after 15 minutes we determined the percentage of cells which failed to attach on the biomaterials because they passed through the pores of the scaffold and adhered to the underlying TCP. Adherent cells were harvested and counted with a Neubauer chamber. As expected, more cells attached to PVDF non-wovens with a denser meshwork. Notably, considerably more cells adhered to non-woven scaffolds with round fibre shape compared to trilobal fibres (n = 3; p = 0.038) indicating that the cross-sectional shape of fibres has impact on MSCs adhesion (Figure 2).

### Proliferation of MSCs in different non-woven scaffolds

**Figure 3:**
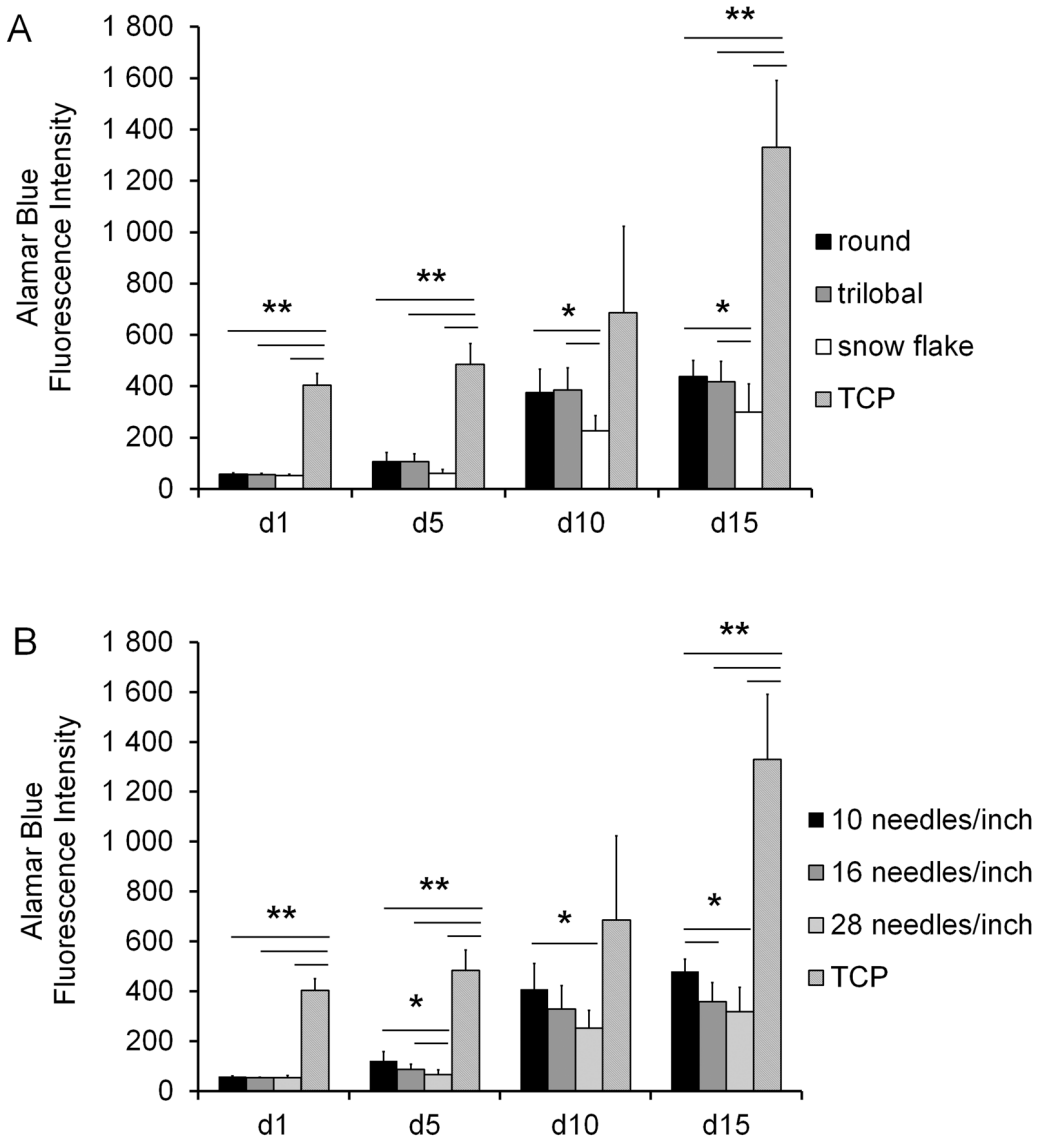
Proliferation rates of MSCs in 3D-PVDF non-wovens. Effect of fibre shape on cell growth was assessed with the Alamar Blue assay 1, 5, 10, and 15 days after seeding. Cells in non-wovens with round or trilobal fibres displayed higher proliferations rates at day 10 and 15. Diagram bars represent the mean value of non-wowens with 10, 16 and 28 needles/inch texturizing (**A**). Effect of different texturizing on cell growth: cells in non-wovens with 10 needles/inch texturizing resulted in the highest proliferation rates at day 5, 10 and 15. Diagram bars represent the mean value of non-wowens with round, trilobal or snowflake fibre shape (**B**; n = 3; *p<0.05; **p<0.005; ***p<0.0005).

To determine whether MSCs are capable of proliferating within PVDF non-wovens we have first tried to harvest the MSCs from the scaffolds after one week of culture by trypsinisation. However, fluorescent microscopic analysis revealed that many MSCs were retained in the PVDF non-wovens despite this procedure. Therefore, we have estimated the proliferation rate using the Alamar Blue assay after 1, 5, 10, and 15 days. Overall, the proliferation rate was significantly higher for MSCs seeded on 2D TCP. Nevertheless, we observed a continuous increase of fluorescence intensity in all PVDF non-wovens indicating that MSCs do proliferate within these scaffolds. Interestingly, at day 10 and 15 the highest proliferation rate was observed on fibres with a round or trilobal cross section rather than on those with snowflake structures (Figure 3A). Furthermore, the proliferation rate was significantly higher in wide-meshed non-wovens (knitted with 10 needles per inch) (Figure 3B).

### MSCs span across the non-woven pores

**Figure 4:**
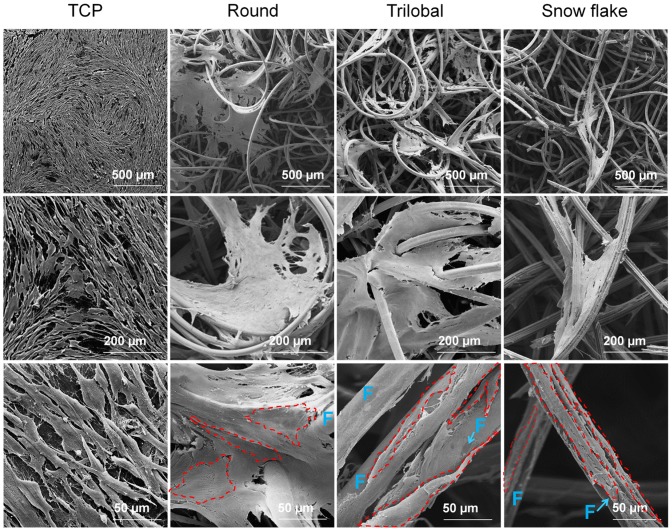
MSC growth on TCP and in 3D-PVDF non-wovens. SEM pictures showing MSC morphology on TCP and in PVDF non-wovens: MSCs span over non-woven pores forming large confluent cell layers. Photos at higher magnifications show that cells align along the fibres and accumulate at fibre intersections. Some individual cells are exemplarily depicted by red dotted lines and PVDF fibres are marked with F.

MSCs growth within the 3D scaffolds was then analysed using scanning electron microscopy (Figure 4). As a general trend, cells aligned along the fibres during their growth and particularly on fibres with snow flake cross section they were arranged in individual fibres. In all non-woven scaffolds cells were preferentially located at the intersection of neighboring fibres. Notably, cell layers span across the non-woven pores displaying cellular sheets with tight cell-cell contacts; thus, in these cellular sheets, single cells are hardly distinguishable in contrast to the well-defined cell layers seen on 2D PVDF controls. Furthermore, we observed that cells seemed to be embedded into some kind of fibrous network, possibly extra-cellular matrix (ECM) proteins. This was observed on TCP and in 3D PVDF non-wovens indicating that the cells contribute to functionalize the scaffold by themselves.

### Differentiation of MSCs within non-woven scaffolds

**Figure 5:**
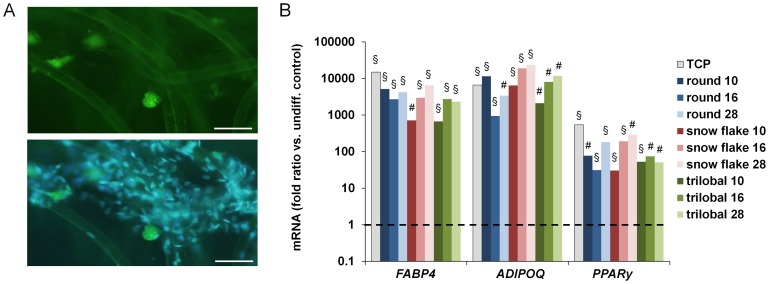
Adipogenic differentiation of MSCs in 3D-PVDF non-wovens. Fluorescence microscopy pictures of MSCs in PVDF non-wovens stained for lipid droplets with BODIPY (green) and nuclei with DAPI (blue) staining (**A**). Adipogenic differentiation was also validated on gene expression level for the adipogenic markers *FABP4*, *ADIPOQ* and *PPARγ*. Gene expression changes are demonstrated in relation to non-differentiated MSCs cultured on the corresponding substrate, indicated by the dotted line (**B**; * P<0.05; # P<0.01; § P<0.001; scale bars  = 100 µm).

Lastly, we analyzed whether MSCs embedded in non-woven structures maintain their *in vitro* differentiation potential. For adipogenic differentiation formation of fat droplets can be observed at single cell level. Such BODIPY positive cells were mainly found on the fibre intersections (Figure 5A). The frequency of differentiated *versus* non-differentiated cells appeared to be similar as compared to TCP control. Expression of adipogenic markers *FABP4*, *ADIPOQ* and *PPARγ* was highly up-regulated as compared to non-differentiated controls and there was no difference between MSC cultured either on TCP or non-wovens (Figure 5B). However, analysis of osteogenic differentiation was hampered by the fact that Alizarin Red staining has high affinity to the PVDF-substrates (Figure S2A) and up-regulation of osteogenic markers was only moderate and not reliable (Figure S2B). This exemplifies, that analysis of *in vitro* differentiation is not trivial in 3D scaffolds. At least adipogenic differentiation potential of MSCs seems to be maintained when cultured on 3D non-woven scaffolds.

## Discussion

The three-dimensional scaffold architecture of a biomaterial has major impact on cellular behavior and cell fate. Non-wovens represent a new concept of biomaterial. Due to their complex 3D conformation they can provide interesting perspectives for some surgical interventions. For example, PVDF non-woven scaffolds might be used as meshes in hernia repair. MSCs are known to mediate the wound healing process by supporting vascularization and by differentiating into many cell types. Studying *in vitro* the interactions between PVDF non-wovens and MSCs may be used to estimate cellularization of the implant also *in* vivo [47].

Polyvinylidene fluoride has been shown to be biocompatible with MSC growth before [48] and this finding was confirmed by our observations. Nonetheless MSCs gained significantly less PDs on PVDF substrates compared to TCP control. Commercially available TCP is usually treated with plasma gas to obtain more hydrophilic surfaces carrying functional groups to facilitate cell attachment. Previous studies reported that argon plasma treated surfaces promoted adhesion and proliferation of various cell types [45], [46], [49], [50]. The results obtained in the present study indicate that the hydrogen peroxide plasma gas used here to treat the PVDF surface does not improve cell adhesion and proliferation on the substrate, but it is conceivable that alternative plasma treatment with argon gas would even further increase cell growth.

Upon initial seeding we observed the highest cell loss in scaffolds with lowest fibre texturizing density (10 needles/inch). These results are in line with the concept that less fibre crimp implies larger pores in the final scaffold and thereby increases the amount of cells falling through it upon seeding [51]. Moreover less fiber crimp leads to fewer fibre-fibre contacts and our results support the notion that cells preferably attach and localise at zones of fibre intersections [52], [53]. The fibre shape hardly affected cellular attachment in scaffolds with high fibre texturizing. However, in scaffolds with larger pores, the round fibre cross sections displayed significantly higher attachment than trilobal and snowflake fibres. Initially, we expected that cells stick better to snowflake structures since they have the largest area and provide a structured surface made of parallel grooves. Considering our results we now hypothesize that surface patterning reduces the area to establish focal adhesions and therefore reduces cellular attachment.

Our results demonstrate that MSCs seeded in 3D-PVDF scaffolds remain viable and proliferate over an extended period of time. The highest proliferation rates were observed between day 5 and day 10, even though they were still much lower than on TCP. It has been reported that MSCs grown in 3D culture systems show a prolonged lag phase of about 5 days before they enter linear growth [54]. Furthermore we observed a correlation between different scaffold porosities and the degree of cell proliferation: fibre texturizing with less needles/inch results in a lower crimp wave length and the larger pores therefore facilitate higher permeability. Hence it is conceivable that the increased cell growth in scaffolds with fibre texturizing of 10 needles/inch result of higher nutrient and oxygen supply.

Scanning electron microscopy pictures revealed that MSCs on 3D-PVDF structures displayed a typical flat, fibroblast like morphology aligned along the fibres. Furthermore, they spanned sheet-like across pores - similar to 2D growth on conventional TCP – which renders it difficult to distinguish single cells. In analogy, previous studies reported that cells seeded in 3D scaffolds form multilayer sheets of cells embedded within a secreted extracellular matrix [55].

Matrix elasticity, surface chemistry and topography are parameters that can direct stem cells differentiation. McBeath *et al*. described how cellular morphology alone regulated commitment of MSCs [56]. Our osteogenic and adipogenic differentiation assays were initially performed using traditional staining methods with Alizarin Red and Oil Red, respectively. However, quantification of colour intensity is hard to assess inside of a complex 3D multi-layered fibrillar biomaterial. Moreover, PVDF showed high affinity for the Alizarin Red and Oil Red. Therefore, we analysed adipogenic differentiation using the green fluorescent BODIPY dye that allowed us to analyse cells at a single cell level. The number of cells with many fat droplets was relatively low but in a similar range as described in previous studies [38]. Overall, adipogenic differentiation of MSCs appeared to be very similar in 3D non-wovens as compared to TCP. Moreover, we did not observed spontaneous adipogenic differentiation of MSCs in non-woven scaffolds in normal culture medium.

## Conclusions

Three-dimensional-PVDF non-woven structures represent a new kind of biomaterial that can be seeded with MSCs. Seeding affinity is higher on round cross sections and smaller pores. MSCs cultivated in 3D-PVDF non-woven scaffolds showed an impaired proliferation capacity but form coherent cell layers bridging the scaffold pores. These results support the notion that PVDF scaffolds are suitable biomaterials for implants in tissue engineering which support MSC growth.

## Supporting Information

Figure S1 **MSC growth on 2D-PVDF surfaces.** MSC were seeded on TCP, PVDF1006 and PVDF1008 (1,000 cells/cm^2^) and population doublings (PDs) within 5 days were estimated by cell counting (**A**). Likewise MSC proliferation was assessed on PVDF1006 substrates after treatment with the oil preparations R641 or PPI and subsequent removal of oil residues with ultrasonic treatment (**B**). Treatment of PVDF substrates with hydrogen peroxide plasma showed no increase in MSC proliferation (**C**).
(TIF)

Figure S2 **Attempts to analyse osteogenic differentiation of MSCs in 3D-PVDF non-wovens.** Upon Alizarin Red staining the PVDF non-wovens were rigorously washed. The image depicts non-specific staining even in non-differentiated controls which hampers reliable quantification (A). Osteogenic differentiation was alternatively estimated by gene expression of *ALP*, *RUNX2*, osteocalcin, and osterix. However, up-regulation of these markers was not reliable and consistent, too (B).
(TIF)
