# Supplementary figures and images for: 3D Non-Woven Polyvinylidene Fluoride Scaffolds: Fibre Cross Section and Texturizing Patterns Have Impact on Growth of Mesenchymal Stromal Cells
Source: PLoS One. 2014 Apr 11;9(4):e94353. doi: 10.1371/journal.pone.0094353 (PMC3984156; doi:10.1371/journal.pone.0094353)

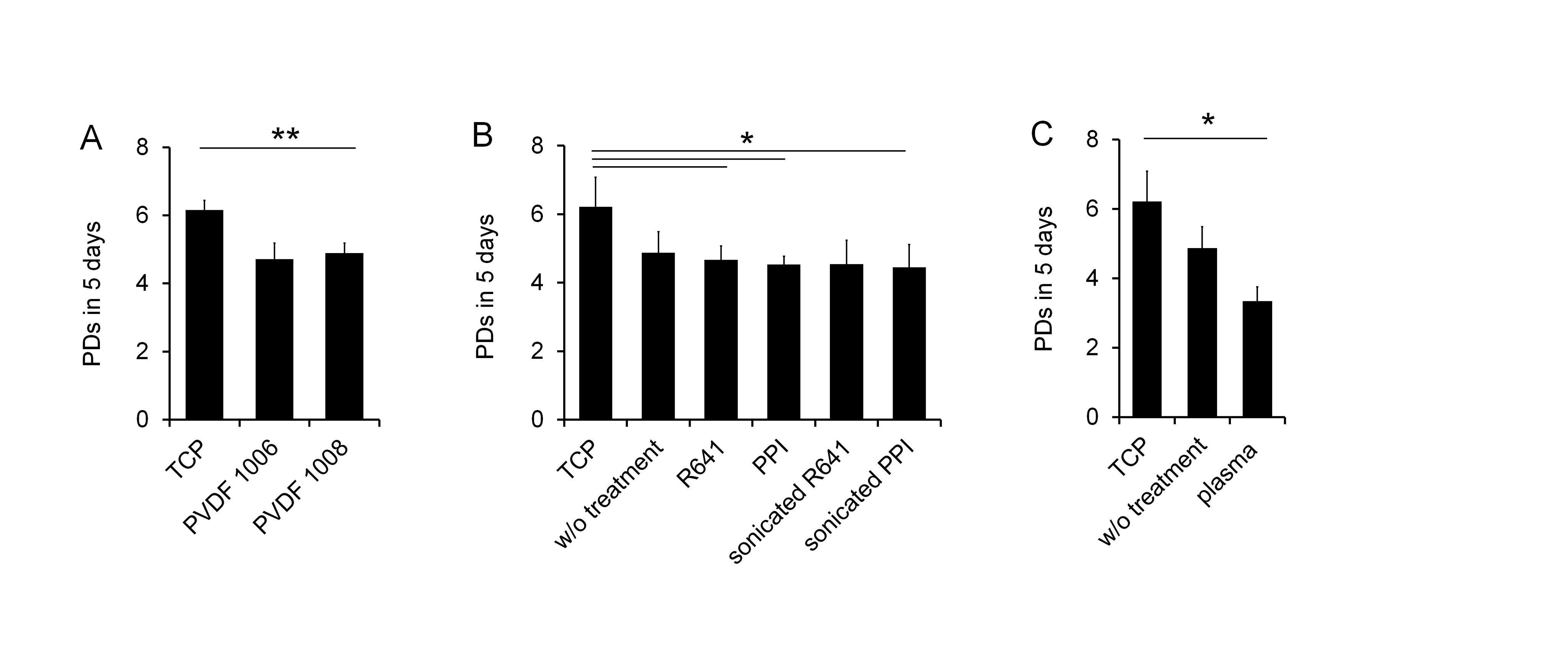

Supplement: Figure S1 — MSC growth on 2D-PVDF surfaces. MSC were seeded on TCP, PVDF1006 and PVDF1008 (1,000 cells/cm2) and population doublings (PDs) within 5 days were estimated by cell counting (A). Likewise MSC proliferation was assessed on PVDF1006 substrates after treatment with the oil preparations R641 or PPI and subsequent removal of oil residues with ultrasonic treatment (B). Treatment of PVDF substrates with hydrogen peroxide plasma showed no increase in MSC proliferation (C). (TIF) [file pone.0094353.s001.tif]

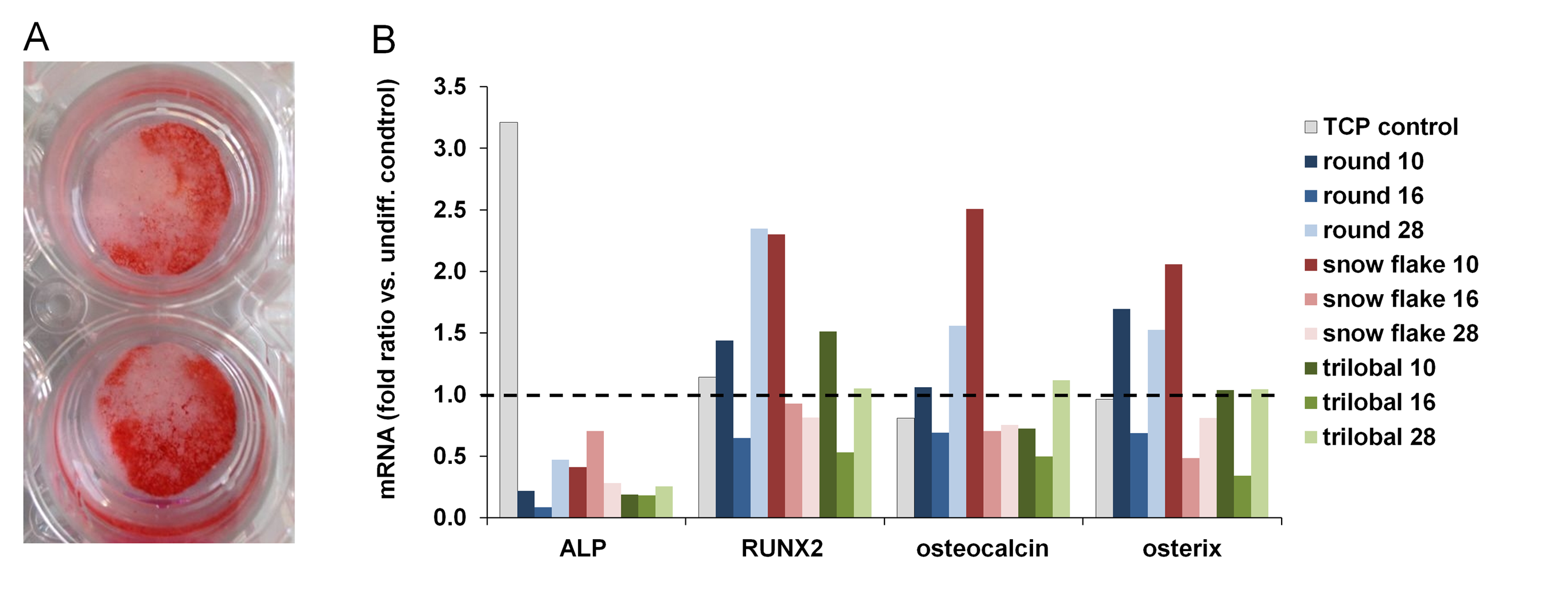

Supplement: Figure S2 — Attempts to analyse osteogenic differentiation of MSCs in 3D-PVDF non-wovens. Upon Alizarin Red staining the PVDF non-wovens were rigorously washed. The image depicts non-specific staining even in non-differentiated controls which hampers reliable quantification (A). Osteogenic differentiation was alternatively estimated by gene expression of ALP, RUNX2, osteocalcin, and osterix. However, up-regulation of these markers was not reliable and consistent, too (B). (TIF) [file pone.0094353.s002.tif]
